# Biocompatible and Biodegradable Nanocarriers for Targeted Drug Delivery in Precision Medicine

**DOI:** 10.3390/biomimetics10070430

**Published:** 2025-07-01

**Authors:** Xin Jin, Hu Qian, Yuxiang Xie, Changzhi Liu, Yuan Cheng, Jinsong Hou, Jiandong Zheng

**Affiliations:** School of Materials and Chemical Engineering, Chuzhou University, Chuzhou 239000, China

**Keywords:** gold nanocages, composite nanospheres, photothermal therapy, targeting delivery, anticancer

## Abstract

Despite the promising natural origin, biocompatibility, and biodegradability of chitosan for biomedical applications, developing biodegradable nanocarriers with controllable sizes and precise drug delivery targeting remains a significant challenge, hindering its integration into precision medicine. To address this, we synthesized gold nanocage (AuNCs)/poly-(N-isopropylacrylamide-co-carboxymethyl chitosan) core-shell multifunctional composite nanospheres (CPAu) through a two-step one-pot method. The resulting CPAu nanospheres (~146 nm in size) exhibited multi-sensitive release properties, excellent biocompatibility, and potent photothermal therapy (PTT) activity. These nanospheres effectively encapsulated diverse antitumor drugs while demonstrating triple responsiveness (thermo-, reduction-, and PTT-triggered) for targeted tumor cell delivery, thereby achieving enhanced antitumor efficacy in combinatorial chemotherapy.

## 1. Introduction

Environmental pollution and population aging are two significant factors contributing to the increasing burden of cancer worldwide. The interplay between these elements exacerbates the risk and prevalence of cancer, making it a formidable public health challenge [1]. Although surgical excision remains a cornerstone of cancer treatment, the integration of chemotherapy, immunotherapy, and innovative delivery systems holds significant promise for addressing residual disease and preventing recurrence. These combined approaches represent a forward-thinking strategy in the ongoing battle against cancer [2,3,4,5]. To address multidrug resistance (MDR) in tumor cells, researchers have developed nanoscale drug delivery carriers—a technology enabling precise medication administration within tumor tissue through controlled release mechanisms [6]. Current clinical-stage nanocarriers (e.g., DOXil^®^, Genexol^®^-PM) demonstrate limited anticancer efficacy due to inadequate tumor penetration, inefficient tumor cell targeting, and suboptimal drug release kinetics [7]. Consequently, multifunctional nanosystems require development with capabilities for tumor tissue recognition and selective therapeutic agent release in response to endogenous pathological stimuli and/or external triggers [8].

Combination therapies leverage the strengths of two modalities to enhance therapeutic efficacy while minimizing side effects. Chemotherapy, a cornerstone of cancer treatment, targets rapidly dividing cells but often suffers from systemic toxicity and drug resistance. Photothermal therapy, on the other hand, utilizes light-absorbing agents to convert near-infrared light into heat, selectively destroying cancer cells with minimal damage to surrounding healthy tissue. Recent advancements in nanotechnology have significantly improved the delivery and effectiveness of chemo-photothermal therapy. For instance, the development of multifunctional nanoparticles that combine photothermal agents with chemotherapeutic drugs has shown promising results in preclinical studies. These nanoparticles can be engineered to target tumor cells specifically, thereby enhancing the accumulation of therapeutic agents at the tumor site and reducing systemic exposure [9,10]. One of the key challenges in photothermal therapy is achieving sufficient light penetration to treat deep-seated tumors. Innovations such as second near-infrared photothermal materials have been developed to address this issue, offering deeper tissue penetration and improved photothermal conversion efficiency [11]. Additionally, the integration of imaging modalities with photothermal therapy allows for real-time monitoring of treatment efficacy and precise targeting of tumor tissues [12]. The combination of chemotherapy and photothermal therapy not only enhances the direct cytotoxic effects on cancer cells but also stimulates an anti-tumor immune response. Studies have demonstrated that this combination can trigger robust anti-tumor immunity, potentially leading to the eradication of both local and distant metastatic tumors [13]. Furthermore, the use of nanocarriers that facilitate the co-delivery of chemotherapeutic agents and photothermal agents can improve the pharmacokinetics and biodistribution of the drugs, enhancing their therapeutic index [14]. In conclusion, the synergistic combination of chemotherapy and photothermal therapy represents a promising strategy for cancer treatment. By leveraging the unique advantages of both modalities, this approach can potentially overcome the limitations of traditional therapies, offering a more effective and less toxic treatment option for patients with cancer [15,16].

Within the nanomaterials spectrum, gold nanostructures including Au nanoflowers, Au nanorods, and Au nanocages are particularly notable due to their exceptional biocompatibility and therapeutic efficacy [17,18,19]. Gold nanocages (AuNCs) are a novel class of nanostructures that have garnered significant attention due to their unique architectural features, including hollow interiors and mesoporous surfaces. These characteristics make AuNCs particularly effective for applications in drug delivery systems, as they facilitate rapid transport of therapeutic agents. The hollow structure of AuNCs allows for high drug loading capacity, while the mesoporous surface enhances the release kinetics of the encapsulated drugs, ensuring efficient delivery to target sites [20]. The versatility of AuNCs extends beyond drug delivery, as they are also employed in photothermal therapy (PTT) due to their excellent photothermal conversion efficiency. This property is leveraged in cancer treatment, where AuNCs can be used to selectively ablate tumor cells upon irradiation with near-infrared light. The ability to conjugate AuNCs with various ligands further enhances their targeting capabilities, allowing for precise delivery to cancerous tissues [21]. In addition to their role in drug delivery and PTT, AuNCs have been integrated into multifunctional nanoplatforms for theranostic applications. These platforms combine diagnostic and therapeutic functions, enabling simultaneous imaging and treatment of diseases. For instance, AuNCs have been used as contrast agents in photoacoustic imaging, providing high-resolution images of tumors and aiding in the precise localization of therapeutic interventions [22]. The design and synthesis of AuNCs are continually evolving, with recent advancements focusing on the control of their structural features to optimize their performance in biomedical applications. Techniques such as the galvanic replacement reaction have been employed to produce AuNCs with tunable optical properties, allowing for their use in a wide range of therapeutic and diagnostic applications [23]. Moreover, AuNCs have been explored in combination with other nanostructures to enhance their functionality. For example, hybrid structures that incorporate AuNCs with other materials have been developed to improve the stability and efficacy of drug delivery systems. These hybrid systems can offer synergistic effects, such as enhanced photothermal and photodynamic therapy capabilities, providing a comprehensive approach to cancer treatment [24]. Overall, the unique properties of AuNCs, including their hollow and mesoporous architecture, make them a promising tool in the field of nanomedicine. Their ability to efficiently deliver drugs, coupled with their potential for use in imaging and therapy, underscores their value in developing advanced therapeutic strategies for various diseases [25].

**Scheme 1:**
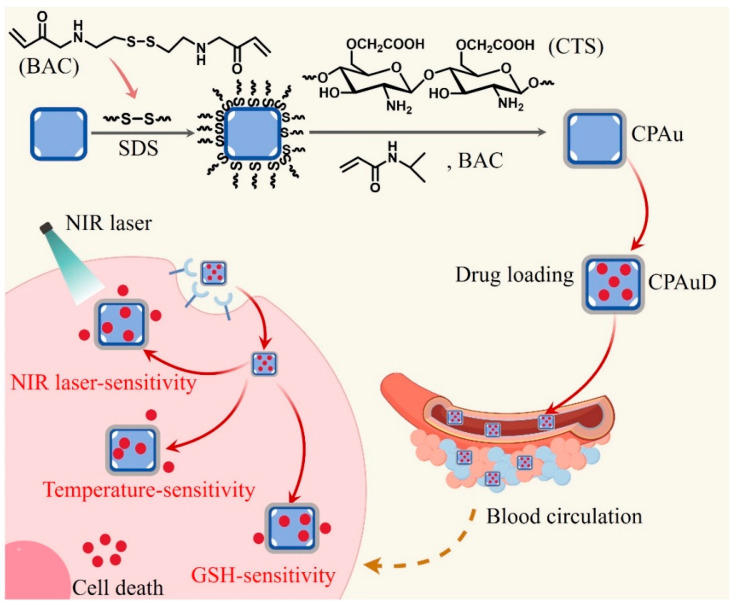
Schematic representation of drug release behavior of DOX-loaded CPAu nanospheres.

Chitosan, a natural polysaccharide derived from chitin, has gained significant attention across biomedical applications due to its unique biocompatibility, biodegradability, and non-toxic properties. In drug delivery systems, chitosan and its derivatives enable the fabrication of nanoparticles, microparticles, and hydrogels capable of encapsulating diverse therapeutic agents, from anticancer drugs and antibiotics to proteins, thereby achieving controlled-release capabilities while enhancing drug stability and bioavailability [26,27]. Concurrently, chitosan serves as an exceptional scaffold material in cell culture applications, promoting cell adhesion and proliferation through its structural resemblance to extracellular matrix glycosaminoglycans; this characteristic makes it ideal for tissue engineering, where chitosan-based scaffolds support osteoblast and chondrocyte differentiation, facilitating bone and cartilage regeneration [28,29]. For bioimaging, chitosan nanoparticles can be functionalized with fluorescent dyes or magnetic particles to enhance visibility in MRI and fluorescence microscopy, enabling non-invasive tracking of drug delivery systems and real-time therapeutic monitoring [30,31]. Therapeutically, chitosan’s inherent antimicrobial properties contribute to wound healing and infection control, with engineered dressings and films forming protective barriers that accelerate healing while preventing microbial invasion. This can be further leveraged through its hydrogel-forming capacity for the development of tissue adhesives and hemostatic agents [32,33]. Furthermore, chitosan’s versatility extends to biomimetics, where materials mimicking natural biological structures are advancing drug delivery, tissue engineering, and regenerative medicine by replicating native tissues’ mechanical and biochemical properties to optimize cellular growth environments [34,35]. Collectively, chitosan’s multifunctionality positions it as a premier biomedical material, with ongoing research addressing limitations and expanding its utility in drug delivery, cell culture, bioimaging, therapy, and biomimetic applications. Herein, we present a novel strategy for fabricating multistimuli-responsive carboxymethyl chitosan (CTS)-based nanospheres as anticancer drug carriers, employing bis(acryloyl)cystamine (BAC)-activated gold nanocages as cores. As depicted in Scheme 1, these core-shell structured Au/P(NIPAM-co-CTS) (CPAu) nanospheres are synthesized through copolymerization of N-isopropylacrylamide and carboxymethyl chitosan onto gold nanocages, forming a poly-(N-isopropylacrylamide-co-carboxymethyl chitosan) (CP) shell. Upon near-infrared (NIR) irradiation, the plasmonic effect of Au nanocages (AuNCs) generates localized heat that triggers a phase transition in the thermosensitive CP copolymer, consequently releasing encapsulated doxorubicin (DOX). These drug-loaded nanocarriers demonstrate significantly enhanced anticancer cytotoxicity via synergistic mechanisms, establishing their potential as versatile nanoplatforms for therapeutic applications (Scheme 1).

## 2. Experimental

### 2.1. Materials

Carboxymethyl chitosan CTS (deacetylation degree > 90%) was purchased from Hefei Bo Biological Co., Ltd. N-Isopropylacrylamide (NIPAM, purity > 98%) was obtained from Tokyo Chemical Co., Ltd. (Tokyo, Japan) and recrystallized before use. AgNO_3_ (>99.8%), sodium dodecyl sulfate (SDS), and HAuCl_4_-3H_2_O (99.9+%) were purchased from Aladdin Reagent Co. Ltd. (Shanghai, China). N, N’-bis(acryloyl)-cystamine (BAC) was obtained from Alfa Aesar Chemicals Co., Ltd. (Karlsruhe, Germany). Polyvinyl pyrrolidone K30 (PVP K30, powder, average Mr 40,000) was purchased from TCI (Shanghai) Development Co., Ltd. (Shanghai, China). Roswell Park Memorial Institute-1640 (RPMI-1640) medium, fetal bovine serum (FBS), Hoechst 33,342, 3-[4, 5-dimethylthiazol-2-yl]-2, 5-diphenyltetrazolium-bromide (MTT), and phosphate-buffered saline (PBS) were provided by Invitrogen (Waltham, MA, USA). All other reagents were of analytical grade and were used as received.

### 2.2. Preparation of Gold Nanocages

AuNCs were synthesized according to a previously reported method [36]. First of all, we placed 60 mL of ethylene glycol (EG) into a 100 mL new flask, adjusted the stirring speed to 260–350 rpm, and preheated it at 150 °C for more than 1 h. Then, 180 μL of an approximately 3 mM Na_2_S solution was added. After waiting for 8–9 min, 10 mL polyvinylpyrrolidone (PVP) (60 mg/mL) was added. Immediately thereafter, we pipetted 0.5 mL AgNO_3_ solution into the flask. The reaction was complete after 10–15 min, at which point, the reaction meniscus was dark ruddy-red while the reaction media appeared green-ochre. Once the reaction had cooled, we span down the product at 10,000 r.p.m for 10 min and dispersed it into 40 mL ultra-pure water. Ag nanocubes of about 27 nm in edge length were prepared as sacrificial templates. Subsequently, AuNCs were prepared through a galvanic replacement reaction between Ag nanocubes and HAuCl_4_. We then pipetted 200 mL of the 9 mM PVP solution into a 500 mL round bottom flask and pipetted 20 mL of the as-prepared Ag nanocubes into the PVP solution. We heated the suspension to a mild boil for approximately 10 min. With a syringe pump, we added a specific volume of the 5 mM HAuCl_4_ solution to the reaction flask at a rate of 0.75 mL per minute. As the HAuCl_4_ solution was being added to the reaction flask, a series of color changes were observed and could be used to estimate the position of the SPR peak for the Au nanocages. The reaction solution was then cooled to room temperature and NaCl was added to saturation. We span down the product at 10,000 r.p.m for 10 min and washed it five times with ultra-pure water. Finally, we stored the Au nanocages in 18.1 MO cm E-pure water until use.

### 2.3. Preparation of the P(NIPAM-CTS) (CP) Nanogels and CPAu Nanospheres

The nanogels were prepared according to the methods reported in previous literature [37]. AuNCs suspensions (1 mL of 0.001 g/mL), SDS (0.0106 g), and BAC (0.028 g) were dispersed in ultrapure water (60 mL) and stirred for overnight (25 °C). Then, under nitrogen degassing, an aqueous solution of NIPAM (3.23 mmol) and CTS (1.77 mmol) was dispersed into the above solution with stirring for 30 min at 70 °C. Next, 1 mL KPS (10 mg/mL) solution was added to the solution to initiate the polymerization. The mixture continued to react at 70 °C for 7 h after dispersion. The obtained mixture was purified by three cycles of centrifuging/water washing, followed by dispersion in water and lyophilization for several days to obtain the CPAu nanospheres.

CP was also synthesized as described above, with the exception that Au was not used.

### 2.4. Measurements

The hydrodynamic size and zeta potential of the nanospheres were characterized using a Zetasizer Nano ZS instrument (Malvern Instruments, Malvern, UK). Hydrodynamic size was determined via dynamic light scattering (DLS), while zeta potential measurements were performed at 25 °C using the Smoluchowski model. This model is appropriate for aqueous dispersions where *κa* > 1 (*κ*: Debye-Hückel parameter; *a*: particle radius). The morphologies of the Au nanocages and nanospheres were examined by transmission electron microscopy (TEM) (Tecnai G20, FEI Tecnai G20, Hillsboro, OR, USA) and scanning electron microscopy (SEM) (Prisma E, JSM7100F, JEOL, Tokyo, Japan). An ultraviolet-visible spectrophotometer was used to measure the absorption spectra of the materials (Lambda 35 CA, PerkinElmer, Waltham, MA, USA). Fourier-transform infrared (FT-IR) spectroscopy analysis was conducted for PNIPAm, CP, and CPAu graft copolymers using an IRSpirit-X spectrophotometer (IRSpirit-X, SHIMADZU, Kyoto, Japan), scanning across the 4000–500 cm^−1^ wavenumber range. Proton nuclear magnetic resonance characterization of the CP graft copolymer was performed on a BRUKER Avance 400 MHz spectrometer (Billerica, MA, USA), employing deuterium oxide as a solvent at ambient temperature, with the residual HDO signal (δ 4.79) serving as internal reference.

### 2.5. Drug Loading Study

Drug encapsulation in the CPAu nanospheres was performed using the physical entrapment method. First, lyophilized CPAu nanospheres (10 mg) were dispersed in 3 mL H_2_O by sonication, followed by dropwise addition of DOX (2 mg/mL) into the above-dispersed nanospheres. Dialysate samples were quantified via UV-vis spectrophotometry at 490 nm to determine nanocarrier capture efficiency through absorbance measurements, with all procedures being conducted under light-restricted conditions. To characterize the intracellular release kinetics, 1 mL of drug-loaded nanomaterials was sealed in dialysis membranes and incubated in PBS buffers under varying physiological parameters, i.e., pH gradients (5.0, 6.5) and GSH concentrations (5 mM, 10 mM), thereby simulating endogenous oxidative stress and lysosomal/extracellular pH environments.

### 2.6. In Vitro Drug Release Study

To examine the drug release properties of the samples, 2 mg CPAu-DOX (CPAuD) nanospheres was dispersed into a dialysis membrane containing 1 mL PBS, which was placed in 30 mL PBS buffer at 25, 37, or 42 °C under a pH value of 7.4, or under near-infrared illumination (808 nm), in the absence and presence of a certain amount of GSH (0 mM, 5 mM, or 10 mM). At pre-selected time intervals, one milliliter of medium was collected and replaced with fresh PBS. The absorbance of the extracted medium was measured using a UV spectrophotometer at a wavelength of 490 nm. The cumulative release (Cur) of doxorubicin was obtained using the following formula:

Cur = Ct/Ctot × 100(1)

where Ct is the cumulative amount of drug released at time t and Ctot represents the total drug contained in the nanospheres used for drug release. All experiments were performed in triplicate.

### 2.7. MTT Assay

An in vitro cytotoxicity assessment of CPAuD against murine mammary carcinoma 4T1 cells was performed via MTT assay. Cells were plated in 96-well microplates at 5 × 10^3^ cells/well density and cultured for 24 h. Following media aspiration, CPAuD-treated solutions were introduced for additional 24-h incubation (DOX concentrations: 0.75, 1.50, 3.00, 6.00, 12.0, 24.0 μM). Subsequently, 20 μL MTT reagent (5 mg mL^−1^) was dispensed into each well and incubated for 4 h. After removing supernatants, 150 μL dimethyl sulfoxide (DMSO) was added to solubilize MTT-formazan crystals. Absorbance at 570 nm with 630 nm reference was quantified (TE2000, Nikon, Tokyo, Japan).cell viability calculated relative to untreated controls.

Cell Viability % = ODe /ODc × 100%(2)

The part of ODeg represented the experimental group, which showed the OD data, whereas the part of ODcg represented the control group. Cytotoxicity assays for free DOX and DOX-loaded nanospheres (CPAuD) were performed using a similar method.

### 2.8. In Vitro Cell Uptake Study

First, 4T1 cells were seeded into a dish at a density of 1.0 × 10^5^ cells/well and cultured in a 37 °C cell incubator for 24 h. Then free DOX and CPAuD were added to each well at the same concentration of 2 μM DOX. The medium was removed after incubation at 37 °C for 4 h or 24 h and the extracellularly ingested drugs or nanospheres were carefully washed out with PBS. Next, 500 μL of serum-free 1640 was added to each well containing 5 μL of Hoechst 33342, incubated at 37 °C for 30 min, and washed with PBS three times. Then, 1 mL of PBS was added and a laser confocal microscope was used for cell observation (TE2000, Nikon, Tokyo, Japan).

## 3. Results and Discussion

Structural confirmation of CP nanogels was achieved through ^1^H NMR and FT-IR analyses. Figure S1 presents the ^1^H NMR spectrum of CP, revealing key structural features: the signal at 3.88 ppm corresponded to the CH_3_COO^−^ group, confirming carboxymethyl chitosan (CMCS) formation. Crucially, the absence of characteristic vinyl proton peaks at 6.12 ppm and 5.62 ppm, coupled with persistent signals at 3.80 ppm and 1.02 ppm (attributed to N-isopropylacrylamide units), provided direct evidence of PNIPAm grafting onto CMCS chains. As shown in Figure S2, FT-IR spectroscopy of CP and CPAu further validated this architecture, with diagnostic absorptions observed at 1715 cm^−1^ (carboxyl groups in chitosan), 1654 cm^−1^ (amide I), 1544 cm^−1^ (amide II), and 2972 cm^−1^ (isopropyl C-H stretching). This spectral dataset collectively confirmed successful PNIPAm grafting onto the chitosan backbone.

**Figure 1:**
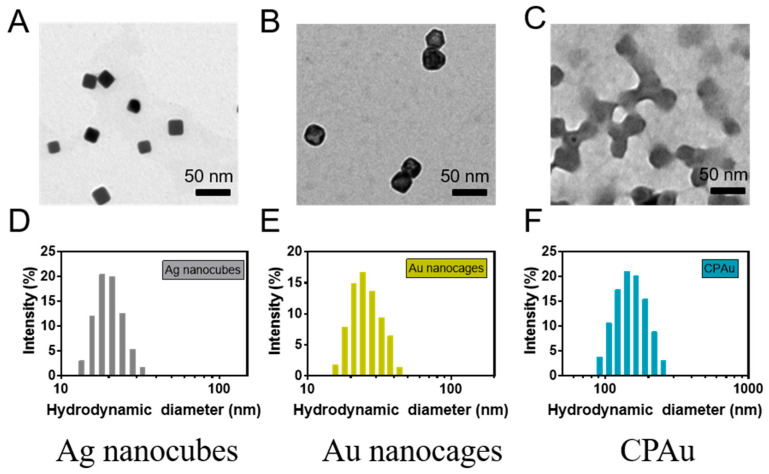
TEM image of (**A**) Ag nanocubes, (**B**) Au nanocages and (**C**) CPAu nanospheres; Hydrodynamic size of (**D**) Ag nanocubes, (**E**) Au nanocages and (**F**) CPAu nanospheres.

A galvanic replacement reaction occurred between the Ag nanocubes and HAuCl_4_, which generated the AuNCs. TEM images of the Ag nanocubes and AuNCs are shown in Figure 1A,B. The hydrated particle size is also shown in Figure 1D,E, which indicates the successful synthesis of gold nanocages. After coating with the copolymer, the nanospheres exhibited an obvious core-shell structure that was mainly distributed within 150 nm (Figure 1C,F). 

Nanospheres have two preponderances: first, they can avoid being detected by the reticuloendothelial system if they are of an effective size; second, they can offer more chances for cell uptake by enhancing the permeability and retention (EPR) effect. For further identification of their stability, the CPAu was then characterized by DLS analysis. As shown in Figure S3, CPAu maintained good physiological stability over 7 days.

**Figure 2:**
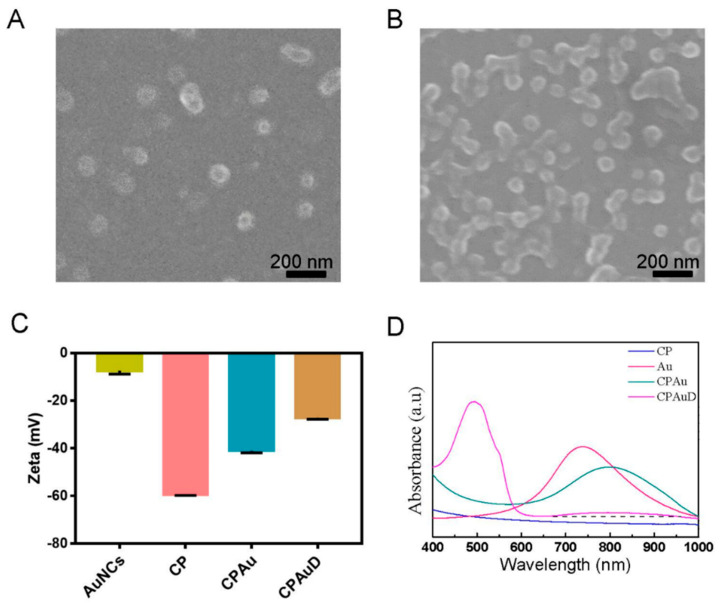
Fe-SEM image of (**A**) CPAu and (**B**) CPAuD; (**C**) Zeta potential of Au, CP, CPAu and CPAuD; (**D**) UV/Vis spectra of CP, AuNCs, CPAu, and CPAuD.

Next, the FE-SEM and the corresponding surface charges of the nanospheres before and after drug loading were measured. As shown in Figure 2A–C, similarly sized nanoparticles exhibited monodisperse properties. TEM analysis revealed the degradation dynamics of nanogels within the tumor microenvironment. The results demonstrated that nanoparticle morphology was disrupted under conditions of 5 mM glutathione (GSH) or pH 6.5, while complete structural disintegration occurred upon reaching 10 mM GSH concentration or pH 5.0, thereby facilitating drug release (Figure S4). The DOX-loaded nanospheres exhibited larger hydrodynamic sizes and less negatively charged surfaces than the corresponding samples, which suggests successful loading of cationic DOX into an anionic nanosphere matrix via strong electrostatic interactions. The characteristic absorption peaks for both Au nanocages and DOX are shown in Figure 2D, which further indicated the successful loading of the drug. Based on the DOX standard curve (Figure S5), the DOX loading efficiency of the CPAuD nanospheres was determined to be 33 ± 4% when the drug concentration was 2 mg/mL. The increase in the ζ-potential of the CPAuD nanospheres is expected to accelerate the intracellular uptake process in cells with a negatively charged surface.

**Figure 3:**
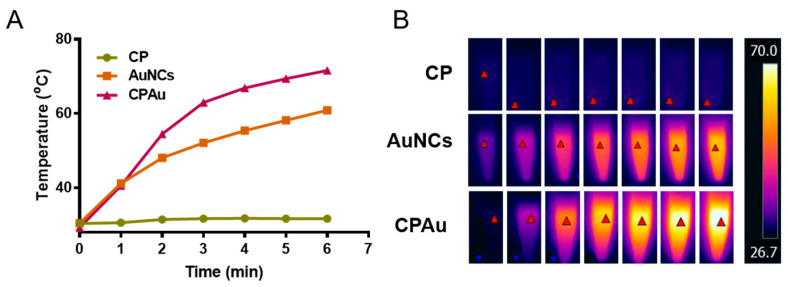
(**A**) Photothermal conversion curves (NIR, 808 nm, 2 W cm^−2^; CP, AuNCs, and CPAu is 40 μg mL^−1^); (**B**) Images taken with thermal imaging of CP, AuNCs, and CPAu aqueous solution.

AuNCs have the physical capability to absorb high NIR light, which provides the opportunity for the construction of remote-controlled carriers sensitive to light. The photothermal conversion efficiency of the carrier is significant for the development of a drug delivery system that responds to light. At a wavelength of 808 nm, CPAu nanospheres and their photothermal effects were investigated. When NIR was irradiated for 6 min, a noticeable increase was observed in AuNCs and CPAu solutions from around 30 °C to 60 °C and 71 °C. As shown by the curve, the CP group’s temperatures barely changed. (Figure 3A). Features that enhanced the photothermal heating of CPAu were generated based on the photothermal conversion performance of CPAu over pure AuNCs. From the thermal images shown in Figure 3B, it was confirmed that this phenomenon was caused by the improvement in colloid stability.

**Figure 4:**
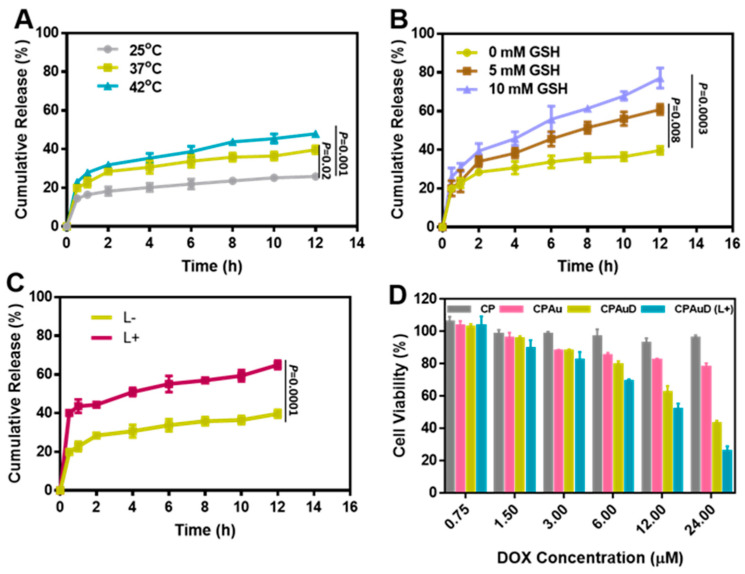
In vitro cumulative release of DOX from CPAuD (**A**) at different temperatures (25, 37, and 42 °C) and (**B**) in the presence and absence of GSH (5 or 10 mM) in PBS buffer at 37 °C; (**C**) The cumulative release of CPAuD after light treatment; (**D**) In vitro cytotoxicity against 4T1 cells of CP, CPAu, CPAuD and CPAuD (L+) (with equivalent DOX concentration, L+: with laser treatment; ±standard deviation, *n* = 5).

The encapsulated drug release process determines the curative effect of anticancer therapy. The encapsulated drug release process is influenced by many factors such as temperature, reducible microenvironment, and lighting effects. To check the temperature sensitivity of the nanospheres in drug delivery, in the condition of a PBS buffer (pH 7.4), we set temperatures of 25, 37, and 42 °C to research the drug-releasing vitality. The data in Figure 4A showthat as the temperature changed from 25 to 42 °C, the CPAuD nanosphere drug release speed increased. The nanospheres displayed a final cumulative drug release of 25.9 ± 1.5%, 39.7 ± 2.0%, and 55.3 ± 3.4% at 25, 37, and 42 °C, respectively. At high temperatures, PNIPAM tends to shrink because of its hydrophobicity [38], and hydrophilic doxorubicin hydrochloride diffuses from the CPAuD nanospheres, leading to a higher speed of the cumulative release process.

It has been reported that some tumor tissues and intracellular sites contain reducing compound-sulfhydryl groups (-SH), resulting in a reducible microenvironment [39,40]. Therefore we should create an environment that must be satisfied for non-reducing and intracellular-mimicking reduction in the presence of 5 or 10 mM GSH in PBS buffer at pH 7.4 in order to investigate the nanosphere drug release behaviors. As shown in Figure 4B, a limited DOX amount (28.4 ± 1.5%) was released from the nanospheres under normal physiological conditions (pH 7.4), whereas the release was accelerated in the presence of 5 mM GSH (33.9 ± 2.6%) or 10 mM GSH (39.4 ± 3.8%) after incubation in PBS for 2 h. The cumulative release of DOX reached 77.1 ± 5.2% in the presence of 10 mM GSH during the release period of 10 h, whereas the samples under normal conditions released a limited amount of DOX drug (39.7 ± 2.0%). The drug release results indicated that the nanospheres exhibited good redox responsiveness for DOX release, which may be beneficial for enhancing intracellular drug delivery upon cellular internalization of the nanospheres.

As shown in Figure 4C, when CPAuD was exposed to light, its cumulative drug release rate was up to 64.9 ± 2.1%, which was higher than that without light treatment. The temperature increased gradually as the NIR light hit, which caused the nanospheres to shrink gradually. Therefore, the observed hydrophilicity was attributed to their hydrophobicity. The importance of light-sensitive carriers is that they can promote release efficiency. In this regard, light-sensitive carriers also provide the prospect of controlled release in a remote manner. Given that CPAuD is a new type of light-sensitive carrier, it could be combined with PTT and chemotherapy to further improve its efficacy in destroying tumor cells.

The antitumor activity and biocompatibility of nanocarriers were rigorously evaluated through MTT assays using breast cancer 4T1 cells cultured under standard conditions. Critical concentration-dependent relationships are documented in Figure 4D. DOX concentrations (0.75, 1.50, 3.00, 6.00, 12.0, 24.0 μM) revealed exceptional biocompatibility of DOX-free formulations (CP/CPAu), maintaining >95% cell viability across all doses (*p* > 0.05). In contrast, DOX-loaded CPAuD exhibited potent cytotoxicity with clear concentration gradients. The half-maximal inhibitory concentration (IC_50_ = 22.5 μM for CPAuD) demonstrated inherent chemotherapeutic efficacy, while NIR-treated CPAuD (IC_50_ = 17.86 μM) achieved a 27.4% lower IC_50_ (*p* < 0.01). This significant enhancement directly correlated with the NIR-triggered photothermal acceleration of drug release (validated in Figure 4C), where localized hyperthermia (∆T > 15 °C at 2.0 mg/mL) potentiated membrane permeability and intracellular DOX accumulation (>48% increase vs. non-irradiated controls). Such concentration–thresholded synergy highlights the therapeutic window between biocompatible carrier doses and tumor-ablative concentrations.

**Figure 5:**
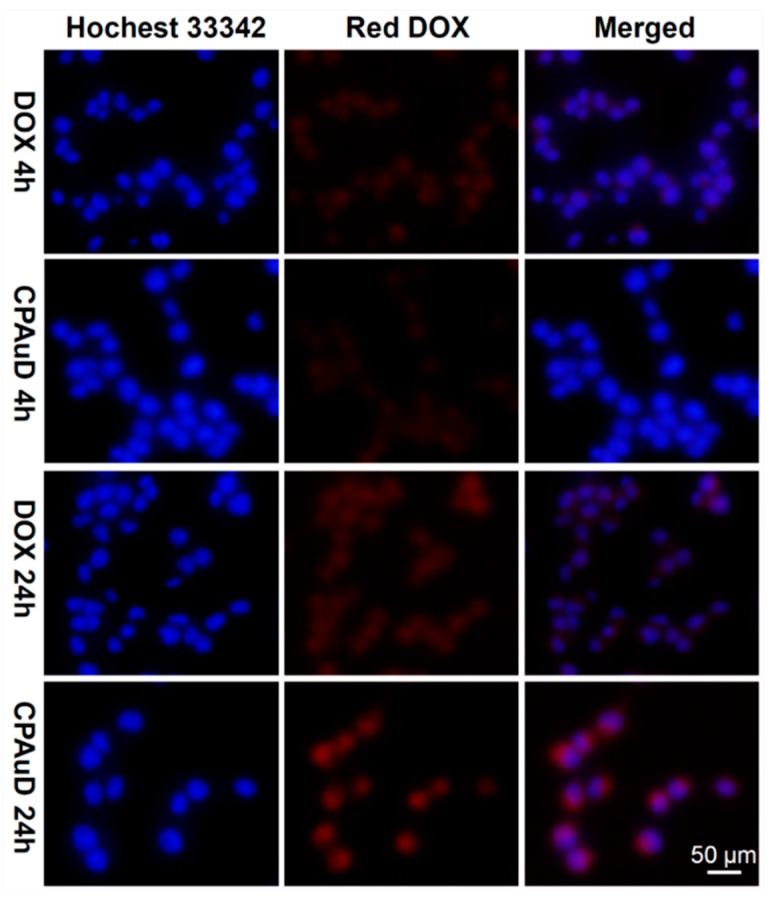
At different periods, free-DOX treated the 4T1 cells image and CPAuD treated the 4T1 cells image (bar scale: 50 μm).

To further study the process of nanosphere uptake by cancer cells, 4T1 cells were treated with different samples for 4 h and 24 h, and their cell morphologies and fluorescence distributions were observed by confocal microscopy. As shown in Figure 5, data from the experiment indicated that in terms of fluorescence, the free DOX sample was more red than the CPAuD sample after 4 h. This was because free DOX and CPAuD have different modes of action: free DOX is passed by free diffusion, while CPAuD is passed by endocytosis. However, the result was exactly the opposite after 24 h; for fluorescence, the CPAuD group was redder than the free DOX group. The comparison indicated that CPAuD could avoid burst-like releases of the drug in a short time; this special feature could be effectively used. The excellent photothermal effects, together with multi-stimulative drug release intelligence, as well as higher intracellular therapeutic accumulation efficiency, are pivotal elements for high antitumor efficacy. Overall, in terms of synergistic chemotherapy and photothermal therapy for improving the tumor therapeutic effect, CPAu was confirmed to be an admirable drug carrier nanoplatform.

## 4. Conclusions

In summary, we designed thermo- and redox-responsive carboxymethyl chitosan (CTS) nanospheres combined with photothermal agents for anticancer therapy. The drug-loaded nanospheres exhibited excellent photothermal performance and controllable size, enabling efficient cellular uptake. Under near-infrared (NIR) light exposure, cancer cells absorbed light and generated heat, triggering nanosphere shrinkage. Subsequently, the reducing intracellular microenvironment induced disulfide cleavage, promoting drug release. Moreover, NIR irradiation further accelerated drug release due to the photothermal-induced structural collapse of nanospheres. This intelligent drug delivery system achieved synergistic cytotoxic effects against cancer cells.

## Data Availability

All data that support the findings of this study are included within the article (and any Supplementary Materials File).

## Supplementary Materials

The following supporting information can be downloaded at: https://www.mdpi.com/article/10.3390/biomimetics10070430/s1, Figure S1: 1H NMR spectra of CP; Figure S2: FT-IR spectra of PNIPAm, CP and CPAu; Figure S3: Hydrodynamic sizes of CPAu evaluated by DLS; Figure S4: TEM images of CPAu nanospheres (0.5 mg/mL) under simulated physiological conditions; Figure S5: Calibration curve between DOX and fluorescein.

## References

[1] Lim E.Y., Kim G.D. (2024). Particulate Matter-Induced Emerging Health Effects Associated with Oxidative Stress and Inflammation. Antioxidants.

[2] Apetoh L., Ladoire S., Coukos G., Ghiringhelli F. (2015). Combining immunotherapy and anticancer agents: The right path to achieve cancer cure?. Ann. Oncol..

[3] Mantovani A. (2009). Inflaming metastasis. Nature.

[4] Steeg P.S. (2016). Targeting metastasis. Nat. Rev. Cancer.

[5] Gerstberger S., Jiang Q., Ganesh K. (2023). Metastasis. Cell.

[6] Wang C., Xu J., Zhang Y., Nie G. (2023). Emerging nanotechnological approaches to regulating tumor vasculature for cancer therapy. J. Control. Release.

[7] Puri S., Mazza M., Roy G., England R.M., Zhou L., Nourian S., Subramony J.A. (2023). Evolution of nanomedicine formulations for targeted delivery and controlled release. Adv. Drug Delivery Rev..

[8] Wang J., Li Y., Nie G. (2021). Multifunctional biomolecule nanostructures for cancer therapy. Nat. Rev. Mater..

[9] Huo J., Jia Q., Huang H., Zhang J., Li P., Dong X., Huang W. (2021). Emerging photothermal-derived multimodal synergistic therapy in combating bacterial infections. Chem. Soc. Rev..

[10] Chen J., Ning C., Zhou Z., Yu P., Zhu Y., Tan G., Mao C. (2019). Nanomaterials as photothermal therapeutic agents. Prog. Mater. Sci..

[11] Xu C., Pu K. (2021). Second near-infrared photothermal materials for combinational nanotheranostics. Chem. Soc. Rev..

[12] Chen Q., Wen J., Li H., Xu Y., Liu F., Sun S. (2016). Recent advances in different modal imaging-guided photothermal therapy. Biomaterials.

[13] Nam J., Son S., Ochyl L.J., Kuai R., Schwendeman A., Moon J.J. (2018). Chemo-photothermal therapy combination elicits anti-tumor immunity against advanced metastatic cancer. Nat. Commun..

[14] Wang Y., Wei G., Zhang X., Huang X., Zhao J., Guo X., Zhou S. (2018). Multistage Targeting Strategy Using Magnetic Composite Nanoparticles for Synergism of Photothermal Therapy and Chemotherapy. Small.

[15] Zhang L., Zhang Y., Xue Y., Wu Y., Wang Q., Xue L., Su Z., Zhang C. (2019). Transforming Weakness into Strength: Photothermal-Therapy-Induced Inflammation Enhanced Cytopharmaceutical Chemotherapy as a Combination Anticancer Treatment. Adv. Mater..

[16] Luo D., Carter K.A., Miranda D., Lovell J.F. (2017). Chemophototherapy: An Emerging Treatment Option for Solid Tumors. Adv. Sci..

[17] Dreaden E.C., Alkilany A.M., Huang X., Murphy C.J., El-Sayed M.A. (2012). The golden age: Gold nanoparticles for biomedicine. Chem. Soc. Rev..

[18] Chen H., Shao L., Li Q., Wang J. (2013). Gold nanorods and their plasmonic properties. Chem. Soc. Rev..

[19] Chow T.H., Li N., Bai X., Zhuo X., Shao L., Wang J. (2019). Gold Nanobipyramids: An Emerging and Versatile Type of Plasmonic Nanoparticles. Acc. Chem. Res..

[20] Chen J., Yang M., Zhang Q., Cho E.C., Cobley C.M., Kim C., Glaus C., Wang L.V., Welch M.J., Xia Y. (2010). Gold Nanocages: A Novel Class of Multifunctional Nanomaterials for Theranostic Applications. Adv. Funct. Mater..

[21] Zhang H., Gao Z., Li X., Li L., Ye S., Tang B. (2021). Multiple-mRNA-controlled and heat-driven drug release from gold nanocages in targeted chemo-photothermal therapy for tumors. Chem. Sci..

[22] Zhan C., Huang Y., Lin G., Huang S., Zeng F., Wu S. (2019). A Gold Nanocage/Cluster Hybrid Structure for Whole-Body Multispectral Optoacoustic Tomography Imaging, EGFR Inhibitor Delivery, and Photothermal Therapy. Small.

[23] Hubert C., Chomette C., Désert A., Madeira A., Perro A., Florea I., Ihiawakrim D., Ersen O., Lombardi A., Pertreux E. (2021). Versatile template-directed synthesis of gold nanocages with a predefined number of windows. Nanoscale Horiz..

[24] Xu Q., Wan J., Bie N., Song X., Yang X., Yong T., Zhao Y., Yang X., Gan L. (2018). A Biomimetic Gold Nanocages-Based Nanoplatform for Efficient Tumor Ablation and Reduced Inflammation. Theranostics.

[25] Wang Y., Miron R.J., Zhang X., Zeng H., Zhang Y. (2024). Nanocages and cell-membrane display technology as smart biomaterials. Periodontol. 2000.

[26] Shariatinia Z. (2019). Pharmaceutical applications of chitosan. Adv. Colloid Interface Sci..

[27] Manna S., Seth A., Gupta P., Nandi G., Dutta R., Jana S., Jana S. (2023). Chitosan Derivatives as Carriers for Drug Delivery and Biomedical Applications. ACS Biomater. Sci. Eng..

[28] Sultankulov B., Berillo D., Sultankulova K., Tokay T., Saparov A. (2019). Progress in the Development of Chitosan-Based Biomaterials for Tissue Engineering and Regenerative Medicine. Biomolecules.

[29] Islam M.M., Shahruzzaman M., Biswas S., Sakib M.N., Rashid T.U. (2020). Chitosan based bioactive materials in tissue engineering applications—A review. Bioact. Mater..

[30] Xiong H.M. (2013). ZnO nanoparticles applied to bioimaging and drug delivery. Adv. Mater..

[31] Gao Y., Wu Y. (2022). Recent advances of chitosan-based nanoparticles for biomedical and biotechnological applications. Int. J. Biol. Macromol..

[32] Youn J., Patel K.D., Perriman A.W., Sung J.S., Patel M., Bouchard L.S., Patel R. (2024). Tissue adhesives based on chitosan for biomedical applications. J. Mater. Chem. B.

[33] Dana P.M., Hallajzadeh J., Asemi Z., Mansournia M.A., Yousefi B. (2021). Chitosan applications in studying and managing osteosarcoma. Int. J. Biol. Macromol..

[34] Zhang Z., Zhang L., Li C., Xie X., Li G., Hu Z., Li S. (2021). Research Progress of Chitosan-Based Biomimetic Materials. Mar. Drugs.

[35] Ahmad M., Manzoor K., Singh S., Ikram S. (2017). Chitosan centered bionanocomposites for medical specialty and curative applications: A review. Int. J. Pharm..

[36] Skrabalak S.E., Au L., Li X., Xia Y. (2007). Facile synthesis of Ag nanocubes and Au nanocages. Nat. Protoc..

[37] Zhang L., Wang L., Guo B., Ma P.X. (2014). Cytocompatible injectable carboxymethyl chitosan/N-isopropylacrylamide hydrogels for localized drug delivery. Carbohydr. Polym..

[38] Ji P., Zhou B., Zhan Y., Wang Y., Zhang Y., Li Y., He P. (2017). Multistimulative nanogels with enhanced thermosensitivity for intracellular therapeutic delivery. ACS Appl. Mater. Interfaces.

[39] Schmaljohann D. (2006). Thermo-and pH-responsive polymers in drug delivery. Adv. Drug Delivery Rev..

[40] Wei H., Cheng S.-X., Zhang X.-Z., Zhuo R.-X. (2009). Thermo-sensitive polymeric micelles based on poly (N-isopropylacrylamide) as drug carriers. Prog. Polym. Sci..

