# Supplementary material for: Biocompatible and Biodegradable Nanocarriers for Targeted Drug Delivery in Precision Medicine
Source: Biomimetics (Basel). 2025 Jul 1;10(7):430. doi: 10.3390/biomimetics10070430 (PMC12292150; doi:10.3390/biomimetics10070430)
Supplement: Supplementary file 1 [file biomimetics-10-00430-s001.zip › biomimetics-3608388-supplementary.pdf]

Supporting Information

Biocompatible and Biodegradable Nanocarriers for Targeted Drug Delivery in Precision Medicine

Xin Jin, Hu Qian, Yuxiang Xie, Changzhi Liu, Yuan Cheng, Jinsong Hou and Jiandong Zheng *

School of Materials and Chemical Engineering, Chuzhou University, Chuzhou 239000, China
* Correspondence: zczhuagong@163.com

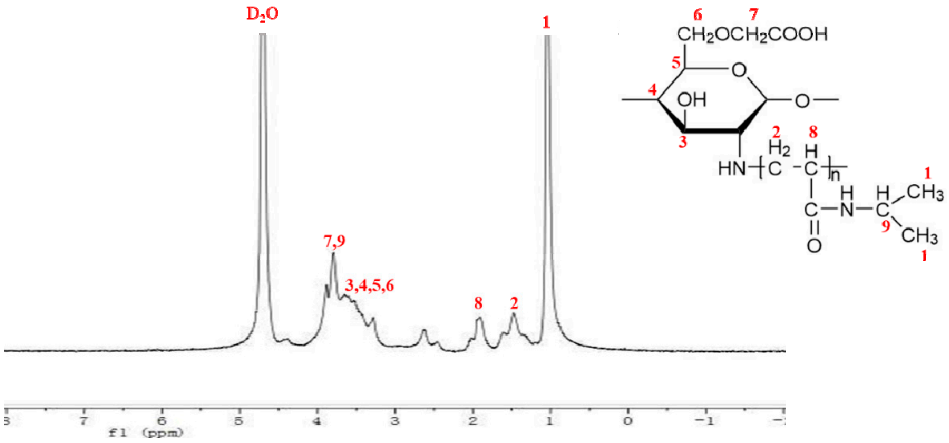

Figure S1. 1H NMR spectra of CP.

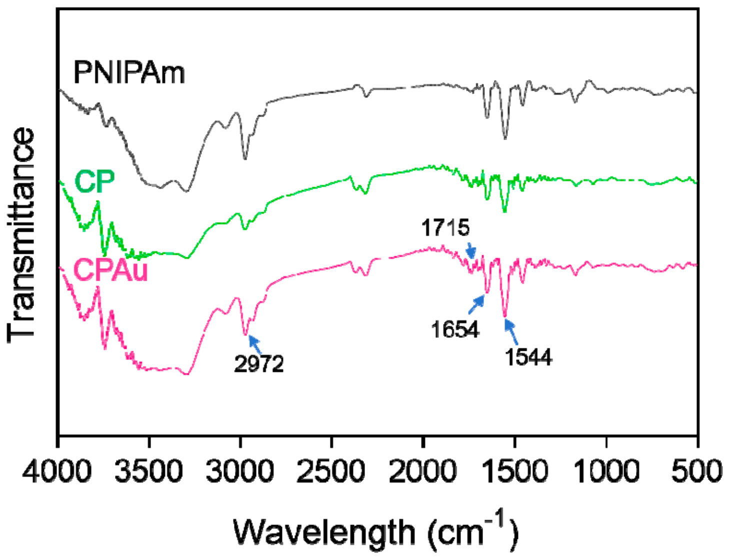

Figure S2. FT-IR spectra of PNIPAm, CP and CPAu.

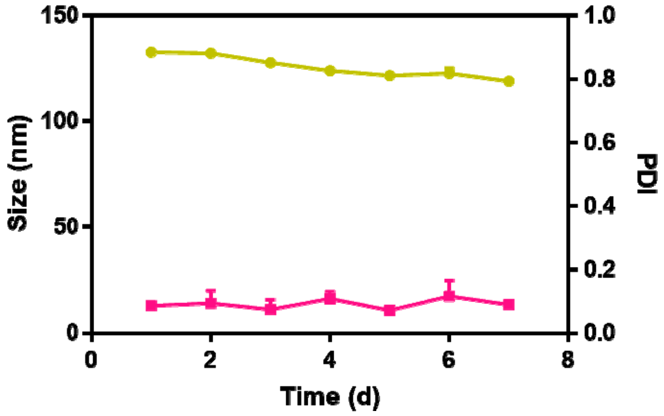

Figure S3. Hydrodynamic sizes of CPAu evaluated by DLS.

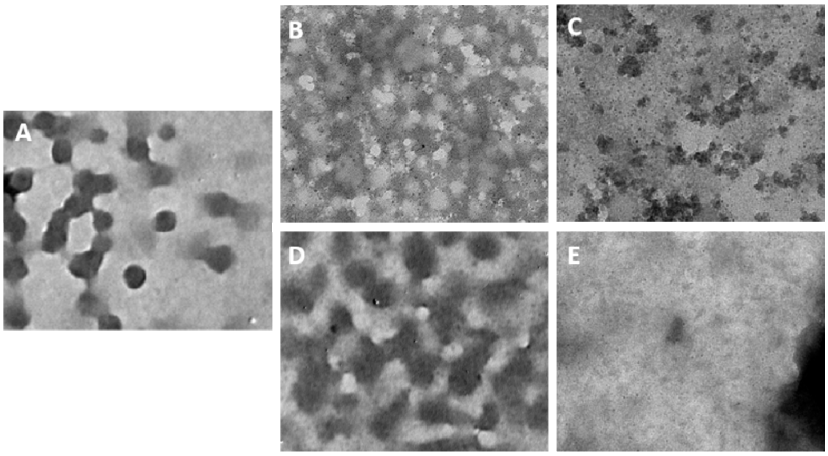

Figure S4. TEM images of CPAu nanospheres (0.5 mg/mL) under simulated physiological conditions: (A) Ultrapure water control; (B) 5 mM GSH; (C) 10 mM GSH; (D) pH 6.5 buffer; (E) pH 5.0 buffer. Samples were incubated at 37°C for 10 min prior to imaging.

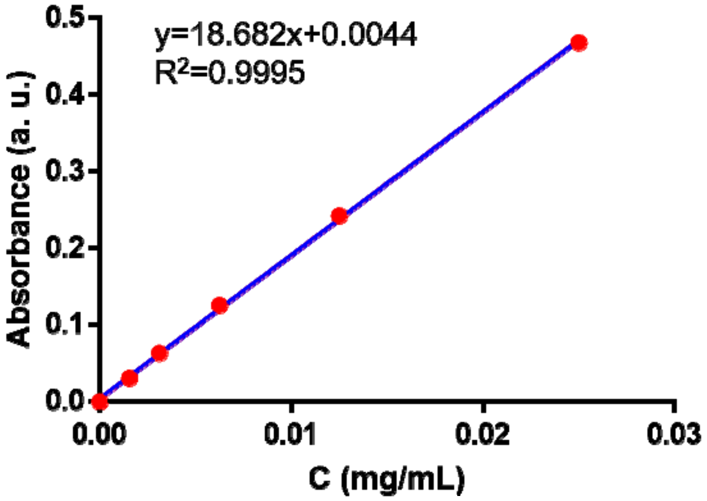

Figure S5. Calibration curve between DOX and fluorescein.
